# Medications Non-adherence Reasoning Scale (MedNARS): Development and psychometric properties appraisal

**DOI:** 10.34172/hpp.2023.26

**Published:** 2023-09-11

**Authors:** Hamid Allahverdipour, Majid Badri, Abdolreza Shaghaghi, Hassan Mahmoodi, Haleh Heizomi, Shayesteh Shirzadi, Mohammad Asghari-Jafarabadi

**Affiliations:** ^1^Research Center of Psychiatry and Behavioral Sciences, Tabriz University of Medical Sciences, Tabriz, Iran; ^2^Health Education & Promotion Department, Tabriz University of Medical Sciences, Tabriz, Iran; ^3^Social Determinants of Health Research Center, Research Institute for Health Development, Kurdistan University of Medical Sciences, Sanandaj, Iran; ^4^Department of Public Health, Neyshabur University of Medical Sciences, Neyshabur, Iran; ^5^Cabrini Research, Cabrini Health, Malvern, VIC, 3144, Australia; ^6^School of Public Health and Preventative Medicine, Faculty of Medicine, Nursing and Health Sciences, Monash University, Melbourne, VIC, 3004, Australia; ^7^Department of Psychiatry, School of Clinical Sciences, Faculty of Medicine, Nursing and Health Sciences, Monash University, Clayton, VIC, 3168, Australia; ^8^Road Traffic Injury Research Center, Tabriz University of Medical Sciences, Tabriz, Iran

**Keywords:** Medication nonadherence, Aged, Surveys and questionnaires, Psychometrics, MedNARS

## Abstract

**Background:**

Proper elucidation of medication non-adherence reasoning especially in older adults might pave the way for an auspicious therapeutic outcome. The main aim of this study was to develop and psychometrically test the Medications Non-adherence Reasoning (MedNARS) questionnaire for application in research and probably practice settings.

**Methods:**

A mixed methods design was utilized to develop the MedNARS. The item pool was mainly generated based on a qualitative query and literature review. The expert panel approved version of the MedNARS was psychometrically assessed on a convenience sample of 220 older patients with chronic disease. The internal consistency, test-retest reliability, content and face validity of the scale were appraised and its construct validity was assed using exploratory and confirmatory factor analyses.

**Results:**

A nine-item version of the MedNARS was drafted based on the classical item analysis procedures and its estimated internal consistency measure of the Cronbach’s alpha (0.85) and test-retest reliability (0.96) were in the vicinity of acceptable range. The exploratory factor analysis (EFA) output revealed a unidimensional structure for the MedNARS and the conducted confirmatory factor analysis (CFA) indicated an acceptable data fit for the extracted one-factor model. The goodness of fit indices were as the followings: χ^2^ /df=1.63(90% CI: 0.02 to 0.11), root mean squared error of approximation (RMSEA)=(0.07), comparative fit index (CFI)=0.95, Tucker–Lewis index (TLI)=0.93 and standardized root mean squared residual (SRMSR)=(0.05).

**Conclusion:**

The study findings were indicative of MedNARS’s applicability and feasibility for use in assessment of medication non-adherence reasoning among the elderly patients with chronic diseases. The MedNARS as a brief and elder-friendly instrument can be applied both in research and practice settings to enhance efficiency, safety, and health outcomes of the therapeutic recommendations.

## Introduction

 The current pace of upsurge in the global aging population and consequent escalation of chronic diseases’ prevalence worldwide, have led to emanation of new challenges for healthcare systems. One of the important dilemmas related to the raised occurrence of chronic disease in old age is acquiescence of the patients to the healthcare providers’ given therapeutic recommendations. Older adults are generally suffer from multi-morbidities therefore, are in added risk of being prescribed with multiple medications.^1^ Polypharmacy is recognized as one of the pivotal patient safety challenge in the world and require a thorough attention of healthcare providers. Medication adherence which refers to a patient’s obedience in taking the prescribed medications in terms of initiation, continuation, and discontinuation^2^ thus, have a consequential impact on improvement or state of health deterioration in chronic diseases sufferers specially in aged patients.

 An extensive heterogeneity was observed in the medication adherence rate in general populations around the world (0-100%)^3,4^ but a part of the discrepancy might be attributed to the application of disparate data collection tools in the implemented studies. A variety of tools have been designed and validated for application in different circumstances.^5^ The introduced instruments for assessment of medication adherence/non-adherence include the Drug Attitude Inventory questionnaire,^6^ the Morisky Medication Adherence Scale,^7^ the eight-item Morisky Medication Adherence Scale (MMAS-8),^8^ the Brief Medication Questionnaire (BMQ), ^9^ the Hill-Bone compliance scale, ^10^ the Medication Adherence Rating Scale (MAR-Scale),^11^ the Brief Adherence Rating Scale^12^ and Clinician Rating Scale.^13^ None of these scales explicitly focus on medications non-adherence reasoning in elder adults.

 Medication adherence by the older adults with type 2 diabetes in Iran was reported to be below 17.7%^14^ and efforts to correct the problematic behavioral pattern was suggested to be not very successful.^15,16^ Relatively identical and a high level of medication non-adherence (61.4%) was reported for Chinese older adults^17^ that represents presence of a cross-border dilemma that need to be expeditiously intervened for prevention of the abrogating consequences.

 The applied instruments for measuring medication adherence/non-adherence amongst Iranian older patients include the short form of the Adherence to Refills and Medications Scale which was used for older adults with chronic disease,^18^ the MMAS-8 among patients with type 2 diabetes,^19,20^ the Treatment Adherence Questionnaire for the Patients with Hypertension.^21^ The best of current knowledge a definite instrument to be applied in studies on older adults for assessment of their medication adherence/non-adherence reasoning was not introduced so far. This is while; importance of paying attention to the older patients’ medication behaviors was emphasized in the scientific literature.^22^

 The main aim of this study therefore, was to develop and psychometrically appraise the Medications Non-adherence Reasoning Scale (MedNARS) as a concise measure for application on old aged populations.

## Materials and Methods

###  Methodology

 An exploratory sequential mixed methods design was used in this study to collect the required data between August to December 2020 in Tabriz, the capital city of East Azerbaijan province, Northwest of Iran.

###  Qualitative phase of the study (concept elicitation) 

 To have an inclusive items pool and concept evocation a qualitative study with a conventional content analysis approach was utilized to recognize the potential items for inclusion in the MedNARS. The study population in this phase consisted of 15 people in the age of 60 or above suffering from at least a chronic disease who had a history of receiving medication prescription for their disease. The study attendees were recruited through a purposive sampling method from a list of free-living registered patients with prescribed medications in Tabriz the capital city of East Azerbaijan province, Northwest of Iran.

 The study data were collected through individual, in-depth, and semi-structured interviews. The interviewees were allowed to determine the time and location of the interview sessions. Each interview began with two main open-ended questions: “What do you think about precipitators or obstacles of medications adherence among elder people?”, “What do you think about important factors that could expedite medications non-adherence in aged people?” The subsequent questions were asked based on the participant’s responses. Each one of the interview sessions lasted approximately 30–60 minutes and interviews were recorded using a voice recorder. Additionally, conventional qualitative content analysis was utilized for data analysis. To increase reliability of the study data all interviews were transcribed verbatim and carefully read several times to prevent misconceptions. The analysis started by identifying units of meaning that were drawn from the transcripts. The codes were allocated in all possible categories to set up a thematic framework for study of medications non-adherence reasoning in elder people.

 The initial item pool for the MedNARS was extracted from the qualitative results (70 codes and 38 statements), and also comprehensive review of existing instruments and scientific literature. Thus, a preliminary draft of the scale was constructed containing eleven items that was sent to an expert panel for feedback and next, based on content validity assessment, nine items were remained in final step.

###  Quantitative phase of the study 

####  Face validity

 To ensure relevancy, clarity and understandability of the MedNARS to the target population subgroup 10 older patients were asked as first step to score the importance of the items on a 5-point Likert scale. The item impact scores (IS) for each item was calculated by multiplying the percentage of people who scored 4 and 5 for an item relative to total number of respondents by the mean score of importance given to that item. The items with impact scores of 1.5 or above were considered satisfactory. Decision about removing or keeping each item was not solely based on the estimated content and face validity indices but also by mutual consensus of the research team members.

####  Content validity

 At the next step, members of an expert panel that included 14 health professions in the field of gerontology, geriatrics, and physicians were asked to assess items of the MedNARS on a three-point Likert scale in terms of importance (where 1 = essential, 2 = useful but not essential, and 3 = not essential) and on a four-point Likert scale in terms of relevance, clarity, and comprehensibility. Minor changes were applied in the items according to the panelists written comments. The values of content validity ratio (CVR) and content validity index (CVI) were also estimated based on the obtained responses.^23,24^ According to the Lawshe’s recommendations, CVR was considered favorable if its estimated value was ≥ 0.51 and a CVI value ≥ 0.79.^23–25^

 The psychometric properties of the MedNARS was investigated through recruitment of 220 aged individuals residing in Tabriz. The exclusion criteria were having a memory or cognitive disorder (such as Alzheimer disease), a serious health condition with severe limiting consequences. To have an adequate sample size for factor analysis inclusion of 20 participants per item was decided.^26^ All efforts were made to choose the study participants based on a maximum variation with regard to their socioeconomic status and from different regions of the study location. A printed copy of the MedNARS was given to the study attendees and they were asked to self-complete the questionnaire.

####  Construct validity 

 To investigate the scale’s construct validity, exploratory factor analysis (EFA) and confirmatory factor analysis (CFA) were performed. The EFA was carried out by the principal component method with direct Oblimin rotation, and the Kaiser–Mayer–Olkin (KMO) and Bartlett’s test were used to assess the model adequacy. In order to demonstrate convergent validity for the MedNARS, a correlation of 0.30 or higher was deemed necessary between each item. Additionally, a successful demonstration of convergent validity was achieved when each item showed a higher correlation with its respective scale compared to the other scales. A correlation of 0.30 or higher between each item of the MedNARS was considered as evidence of convergent validity. A higher correlation of each item with its scale than with the other scales was considered a successful demonstration of convergent validity. Stewart and Ware recommended that items scoring less than 0.30 should be removed from the analysis.^27^ It was decided to apply oblique rotation if the oblique factor correlation matrix displays correlations of 0.30 or above.

 The EFA and CFA were performed to examine the construct validity of the MedNARS, utilizing two subsamples of 95 and 125, respectively. The model fitness was determined using the χ^2^/df < 5, the root mean square error of examination (RMSEA) < 0.08, standardized root mean square residual (SRMSR), the comparative fit index (CFI) > 0.9, Tucker-Lewis Index (TLI). The robust maximum likelihood estimation method was used for CFA and asymptomatic covariance matrix was considered a weighted matrix in the analysis. The study data analysis was performed using IBM SPSS Statistics 21.0 (IBM SPSS Statistics, ARMONK, USA) and the STATA 14 (Stata Corp, College Station, TX). *P* values less than 0.05 were considered as significant.

####  Internal consistency and reliability of the MedNARS

 The internal consistency of the translated scale was assessed using Cronbach’s alpha, and McDonald’s omega total. McDonald’s omega ranged from 0.75 to 0.96.^28,29^ The Cronbach’s alpha as a measure of and intra-class correlation coefficient (ICC) were estimated and values of 0.70 or above for Cronbach’s alpha was deemed to be satisfactory and values in the range of 0.61–0.80 and above 0.80 were considered as being good and excellent for ICC.^30^

####  The MedNARS scoring guideline

 The MedNARS measures medication non adherence reasoning through items with a 5-point Likert scale, where 1 refers to frequently, 2 = Usually, 3 = Occasionally, 4 = Rarely, and 5 refers to Not at all, so higher scores represent higher levels of medication adherence.

## Results

 The mean age of the participants (n = 220) was 68.2 (SD: 7.2), ranging from 61 to 75.4 years. The majority of the participants were women (57.73%), single/divorced/widowed (66.36%), and unemployed (62.73%), and also about 32.73% of the participants had elementary and secondary level of literacy level. Table 1 provides detailed information on the demographic characteristics of the participants in the quantitative phase.

**Table 1:** Demographic and underlying characteristics of the participants (N = 220)

**Variables**	**No. (%)**
Gender	
Male	93 (42.2)
Female	127 (57.7)
Literacy level	
Illiterate	56 (25.4)
Elementary	72 (32.7)
Secondary school	46 (20.9)
Diploma	42 (19.0)
University	4 (1.8)
Marital Statues	
Married	74 (33.6)
Single/divorced/widowed	146 (66.3)
Employment	
Unemployed	138 (62.7)
Employed	82 (37.2)
Health Insurance	146 (66.3)
Insured	74 (33.6)
Uninsured	146 (66.3)
Residence status	
Personal house	92 (41.8)
with their children or family members	129 (58.6)

###  Content and face validity

 Based on the results of CVR and CVI, the estimated CVR for two items (4 and 5) was lower than 0.6. As a result, we removed these items from the main questionnaire. In the assessment of face validity using the impact score method, no item was scored lower than 1.5 per item therefore they were remained on the scale. Next, we revised final items based on the recommendations of the expert panel (Table 2).

**Table 2:** The scores of CVI, CVR and IS for items

**Item**	**Item content**	**CVI**	**CVR**	**IS**	**Result**
1	Have you ever stopped taking your prescribed medications due to experiencing their side effects?	1	1	4	corelate with qualitative recommendations of expert panel and confirmation of research team
2	Have you ever stopped taking your medications due to prescription of their long-term use?	1	0.8	4	Accept without change
3	Have you ever stopped taking your prescribed medications due to ineffectiveness of your therapeutic regimen?	1	1	4	corelate with qualitative recommendations of expert panel and confirm the research team
4	Have you ever stopped taking your medications due to prescription of multiple drug use at the same time?	1	1	4	Accept without change
5	Have you ever stopped taking your prescribed medications due to their interactions?	1	0.8	4	corelate with qualitative recommendations of expert panel and confirm the research team
6	Have you ever stopped taking your prescribed medications due to their taste, size or form?	1	1	4	Accept without change
7	Have you ever stopped taking your prescribed medications due to your illness severity or symptoms?	1	1	4	corelate with qualitative recommendations of expert panel and confirm the research team
8	Have you ever stopped taking your prescribed medications due to the impacts of your illness on your life?	0.9	0.8	3.6	corelate with qualitative recommendations of expert panel and confirm the research team
9	Have you ever stopped taking your medicines due to being prescribed with multiple medications?	1	1	4	Accept without change

Abbreviations: CVR, content validity ratio; CVI, content validity index; IS, impact score.

###  Construct validity

 The KMO measure of sampling adequacy was 0.89 and the sampling adequacy was confirmed by the Bartlett’s test of sphericity (*P* < 0.001). The total estimated variance explained was 59.20 %. The scale’s factor loadings are shown in Table 3.

**Table 3:** Model adequacy and factor loadings of the scales

	**Items**	**F**
1	M1	0.87
2	M7	0.74
3	M4	0.74
4	M8	0.72
5	M2	0.71
6	M5	0.70
7	M3	0.66
8	M6	0.66
9	M9	0.61

**Figure 1:**
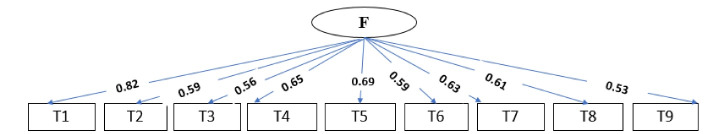
Factor model for the MedNARS scale obtained from Confirmatory analysis

**Figure 2:**
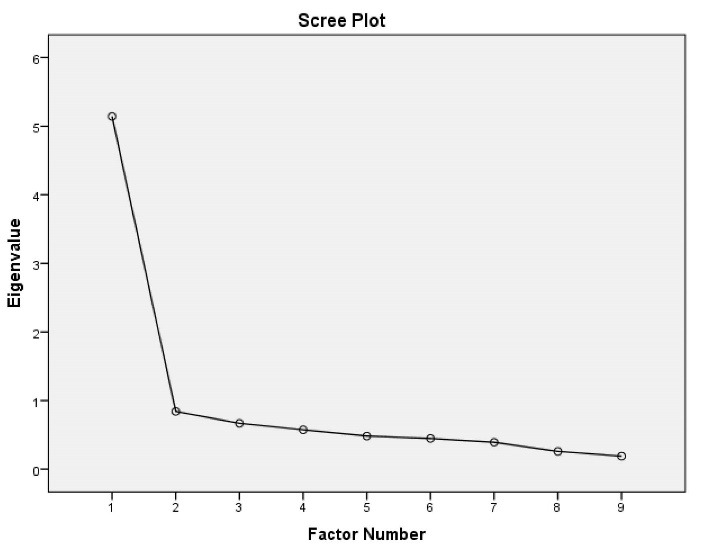
Scree plot for the exploratory factor analysis of the MedNARS.

 We conducted CFA to assess how well the EFA-extracted model fits the observed data (Figure 1). The best model of fit was obtained by applying covariance matrixes and measuring fit indices. As shown in Figure 1, all the fit indices were satisfactory. The CFA on the 9 items yielded the following results: (χ^2^/df = 1.63, RMSEA = 0.07 (90% CI: 0.02 to 0.11), CFI = 0.95, TLI = 0.93 and SRMSR = (0.05) that indicate an acceptable fit of the proposed model. Therefore, the final model can be used to confirm the questionnaire’s factors. In addition, all item-scale relationships and the correlation among the scales were all significant (*P* < 0.05). The scree plot also suggested unidimensionality of the MedNARS (Figure 2).

###  Reliability

 The estimated values for internal consistency measure of Cronbach’s alpha (α = 0.86) and McDonald’s Omega = 0.87(95% CI: 0.84 to 0.89) and test–retest analysis of the scale’s stability over time (ICC = 0.85, *P* = 0.000), were in the vicinity of acceptable range.

## Discussion

 The main objective of this study was to develop and psychometrically appraise a new tool for assessing medication non-adherence reasoning (MedNARS) among elderly patients with chronic diseases. The study found that the developed scale, called the MedNARS, can be considered potentially a suitable tool for use in assessing medication adherence/non-adherence among older people. The MedNARS includes items that focus on the main reasons of medication non-adherence, such as experiencing their adverse effects, being prescribed by long-term medications, having a sense of taking non-effective medication, having difficulties with multi-medications and forms of prescribed drugs, severity of the current disease and its symptoms, and quandary of taking multiple dosages per day. These findings suggest that patients’ medication adherence/non-adherence especially among elderly people could be dependent on multi-layered factors but aged people individualistic experiences and circumstances should be considered carefully as more paramount influencing parameters of their therapeutic behaviors.^31^ The values of MedNARS’s reliability, face, content, and construct validities were indicative of its potential applicability in studies of medication non-adherence among older people. Application of the Cronbach’s alpha^32^ and McDonald’s Omega^33^ indices was to assure validity of the scale’s reliability interpretation.^28,34^

 Measurement tools for medication adherence/non-adherence can be classified in five groups: those that assess only knowledge of the potential respondents about medication-taking behavior, scales that focus on the knowledge about medication-taking behavior and its barriers, instruments that concentrate solely on barriers to medication adherence, those that assess only beliefs associated with medication adherence, and finally tools that collect data about the respondents’ perceived barriers regarding medication adherence.^35^ The MedNARS concentrate on the reasoning of the patients to disobey prescribed therapeutic regimen Understanding these subjective or objective argumentation about medication non-adherence could shed light on pathways that may mediate the process of medication adherence in elder people. Hence, planning and implementation of tailored made and feasible therapeutic interventions will be possible.

 The final version of the MedNARS included nine items, indicating its user-friendliness for older adults and ease of use in practice settings. The common suggested reasons for medication non-adherence in the literature include number of prescribed drugs, their adverse effects, and their type of administration among elderly patients.^36^ The MedNARS including all these parameters and focuses on extending all possible reasoning of medication non-adherence among alder people to help better understanding of the factors that might impede successful treatment of diseases. Thus, MedNARS is comparable with the MAR-Scale and the BMQ^9,37^ in providing a full picture of dilemma healthcare providers are facing in practice setting to attract sufficient cooperation of the patients with their medication regimen. The applied phases of the study in preparation of the MedNARS imply its comprehensiveness in provision of such a portrayal to be applied for evidence informed interventions.

## Limitations

 While there were a number of strengths of the current study, including the applied sample size that satisfies the statistical standards some methodological limitations need to be considered in interpretation of the findings. First, intentionally recruitment of sample from elder people without intellectual disability or co-occurring major psychiatric disorders, limit the generalization of the findings to those with co-occurring neurodevelopmental disabilities (e.g., intellectual disability, attention-deficit disorder) or psychiatric disorders (e.g., anxiety disorders, psychotic disorders) as reported in other psychometric studies.^38^ Cross-sectional nature of the study also prevented assessment of the temporality of the therapeutic adherence/non-adherence behaviors of the studied patients over time.^39^ The applied self-report data collection procedure might also have introduced a social desirability bias with influence on the participants’ response pattern.^40^ Fourth, the researchers were unable to perform subgroup analyses to check if sex or gender moderates the psychometric properties of the MedNARS. Use of a convenience sample and not performing concurrent and discrimination validity appraisal due to logistic restrains also warrant further speculation in future studies.

 A sizeable multicenter study will be worthwhile in future to boost sample representativeness and external validity of the findings. Cross-cultural validation studies are also required across different socio-demographic and lifestyle groups of the aged population. Future research will need to empirically disentangle all these caveats for proving cross-cultural applicability of the MedNARS in diverse contextual backgrounds.

## Conclusion

 The aforementioned findings clearly revealed that the MedNARS (Supplementary File 1) with nine-items can be used to assess medication non-adherence reasoning among the elderly patients with chronic diseases. To the best of current knowledge, this is the first instrument for measuring medication non-adherence rationales of elder patients with chronic disease in Iran. The MedNARS as a brief and elder-friendly instrument can be applied in practice and research settings for assessing medication non-adherence in a wide range of population sub-groups that might be prescribed by contrasting therapeutic regimens in similar circumstances and diseases. Future cross-cultural research is recommended to assess the applicability of the MedNARS in diverse populations of the aged patients with dissimilar illnesses. Construction and psychometric analysis of the MedNARS was one of the important steps in introducing a valid and applicable instrument for use in research and practice settings to enhance efficiency, safety, and health outcomes of the therapeutic recommendations.

## Acknowledgments

 We sincerely thank all the patients for their participation in this study.

## Competing Interests

 Hamid Allahverdipour is the editor-In-chief, Abdolreza Shaghaghi and Mohammad Asghari-Jafarabadi are also associate editors of the Health Promotion Perspectives. Other authors declare no conflicts of interest in performing this research and preparation of the final report. 

## Ethical Approval

 Ethical approval for the study protocol was obtained from the Medical Ethics Board of Trustees (MEBoT) affiliated to the Tabriz University of Medical Sciences (IR.TBZMED.REC.1396.435) and all the participants voluntarily signed an informed consent form to participate in the study before the interviews and data collection stage.

## Funding

 The study was founded by the Tabriz University of Medical Sciences, Iran.

## Supplementary Files


Supplementary file 1. The Final Medications Non-adherence Reasoning Scale (MedNARS) and scoring guideline.

## References

[1] Félix IB, Henriques A (2021). Medication adherence and related determinants in older people with multimorbidity: a cross-sectional study. Nurs Forum.

[2] Larsen RE, Pripp AH, Krogstad T, Johannessen Landmark C, Holm LB (2022). Development and validation of a new non-disease-specific survey tool to assess self-reported adherence to medication. Front Pharmacol.

[3] Horne R, Cooper V, Wileman V, Chan A (2019). Supporting adherence to medicines for long-term conditions. Eur Psychol.

[4] Nieuwlaat R, Wilczynski N, Navarro T, Hobson N, Jeffery R, Keepanasseril A (2014). Interventions for enhancing medication adherence. Cochrane Database Syst Rev.

[5] Lam WY, Fresco P (2015). Medication adherence measures: an overview. Biomed Res Int.

[6] Hogan TP, Awad AG, Eastwood R (1983). A self-report scale predictive of drug compliance in schizophrenics: reliability and discriminative validity. Psychol Med.

[7] Morisky DE, Green LW, Levine DM (1986). Concurrent and predictive validity of a self-reported measure of medication adherence. Med Care.

[8] Morisky DE, Ang A, Krousel-Wood M, Ward HJ (2008). Predictive validity of a medication adherence measure in an outpatient setting. J Clin Hypertens (Greenwich).

[9] Svarstad BL, Chewning BA, Sleath BL, Claesson C (1999). The Brief Medication Questionnaire: a tool for screening patient adherence and barriers to adherence. Patient Educ Couns.

[10] Kim MT, Hill MN, Bone LR, Levine DM (2000). Development and testing of the hill-bone compliance to high blood pressure therapy scale. Prog Cardiovasc Nurs.

[11] Thompson K, Kulkarni J, Sergejew AA (2000). Reliability and validity of a new Medication Adherence Rating Scale (MARS) for the psychoses. Schizophr Res.

[12] Byerly MJ, Nakonezny PA, Rush AJ (2008). The Brief Adherence Rating Scale (BARS) validated against electronic monitoring in assessing the antipsychotic medication adherence of outpatients with schizophrenia and schizoaffective disorder. Schizophr Res.

[13] Kemp R, Hayward P, Applewhaite G, Everitt B, David A (1996). Compliance therapy in psychotic patients: randomised controlled trial. BMJ.

[14] Ranjbaran S, Shojaeizadeh D, Dehdari T, Yaseri M, Shakibazadeh E (2020). Determinants of medication adherence among Iranian patients with type 2 diabetes: an application of health action process approach. Heliyon.

[15] Bastani P, Bikineh P, Mehralian G, Sadeghkhani O, Rezaee R, Kavosi Z (2021). Medication adherence among the elderly: applying grounded theory approach in a developing country. J Pharm Policy Pract.

[16] Samadi S, Bayat A, Taheri M, Joneid BS, Roozbahani N. Knowledge, attitude and practice of elderly towards lifestyle during aging. J Inflamm Dis 2007;11(1):83-4. [Persian].

[17] Cao W, Abdul Kadir A, Wang J, Hu L, Wen L, Yu M (2022). Medication non-adherence and associated factors among older adult stroke survivors in China. Front Pharmacol.

[18] Barati M, Taheri-Kharameh Z, Bandehelahi K, Yeh VM, Kripalani S (2018). Validation of the Short Form of the Adherence to Refills and Medications Scale (ARMS-SF) in Iranian elders with chronic disease. J Clin Diagn Res.

[19] Moharamzad Y, Saadat H, Nakhjavan Shahraki B, Rai A, Saadat Z, Aerab-Sheibani H (2015). Validation of the Persian version of the 8-Item Morisky Medication Adherence Scale (MMAS-8) in Iranian hypertensive patients. Glob J Health Sci.

[20] Ghanei Gheshlagh R, Ebadi A, Veisi Raygani AK, Nourozi Tabrizi K, Dalvandi A, Mahmoodi H. Determining concurrent validity of the Morisky Medication Adherence Scale in patients with type 2 diabetes. Iran J Rehabil Res Nurs 2015;1(3):24-32. [Persian].

[21] Dehghan M, Dehghan-Nayeri N, Iranmanesh S (2016). Translation and validation of the Persian version of the treatment adherence questionnaire for patients with hypertension. ARYA Atheroscler.

[22] Furr RM. Scale Construction and Psychometrics for Social and Personality Psychology. SAGE Publications Ltd; 2011. 10.4135/9781446287866

[23] Liamputtong P. Handbook of Research Methods in Health Social Sciences. Springer; 2019. 10.1007/978-981-10-5251-4

[24] Polit DF, Beck CT, Owen SV (2007). Is the CVI an acceptable indicator of content validity? Appraisal and recommendations. Res Nurs Health.

[25] Cottrell RR, McKenzie JF. Health Promotion & Education Research Methods: Using the Five Chapter Thesis/Dissertation Model. Jones & Bartlett Publishers; 2010.

[26] Mundfrom DJ, Shaw DG, Ke TL (2005). Minimum sample size recommendations for conducting factor analyses. Int J Test.

[27] Kwok C, Fethney J, White K (2010). Chinese breast cancer screening beliefs questionnaire: development and psychometric testing with Chinese-Australian women. J Adv Nurs.

[28] Beland S, Cousineau D, Loye N (2017). Using the Mcdonald’s omega coefficient instead of Cronbach’s alpha. McGill J Educ.

[29] Gignac GE, Reynolds MR, Kovacs K (2019). Digit span subscale scores may be insufficiently reliable for clinical interpretation: distinguishing between stratified coefficient alpha and omega hierarchical. Assessment.

[30] Bartko JJ (1966). The intraclass correlation coefficient as a measure of reliability. Psychol Rep.

[31] McHorney CA, Gadkari AS (2010). Individual patients hold different beliefs to prescription medications to which they persist vs nonpersist and persist vs nonfulfill. Patient Prefer Adherence.

[32] Thorndike RM (1995). Thorndike RMBook Review: Psychometric Theory (3rd ed) by Jum Nunnally and Ira Bernstein New York: McGraw-Hill, 1994, xxiv + 752 pp. Applied Psychological Measurement.

[33] McDonald RP. Test Theory: A Unified Treatment. New York: Psychology Press; 2013. 10.4324/9781410601087

[34] Vaske JJ, Beaman J, Sponarski CC (2017). Rethinking internal consistency in Cronbach’s alpha. Leis Sci.

[35] Nguyen TM, La Caze A, Cottrell N (2014). What are validated self-report adherence scales really measuring?: a systematic review. Br J Clin Pharmacol.

[36] Gellad WF, Grenard JL, Marcum ZA (2011). A systematic review of barriers to medication adherence in the elderly: looking beyond cost and regimen complexity. Am J Geriatr Pharmacother.

[37] Unni EJ, Olson JL, Farris KB (2014). Revision and validation of Medication Adherence Reasons Scale (MAR-Scale). Curr Med Res Opin.

[38] Chang JC, Lai MC, Chien YL, Cheng CY, Wu YY, Gau SS (2023). Psychometric properties of the Mandarin version of the autism diagnostic observation schedule-generic. J Formos Med Assoc.

[39] Asgeirsdottir BB, Huffhines L, Sigurvinsdottir R, Wherry JN (2021). Dyadic reports using the parental support after child sexual abuse measure: psychometrics and associations with post-traumatic stress disorder symptoms. Child Abuse Rev.

[40] Hojat M, DeSantis J, Cain RA, Speicher MR, Bragan L, Calabrese LH (2021). Attitudes toward osteopathic medicine scale: development and psychometrics. Int J Med Educ.

