# Corrigendum to: Medications Non-adherence Reasoning Scale (MedNARS): Development and psychometric properties appraisal

**DOI:** 10.34172/hpp.025.44568

**Published:** 2025-05-06

**Authors:** Hamid Allahverdipour, Majid Badri, Abdolreza Shaghaghi, Hassan Mahmoodi, Haleh Heizomi, Shayesteh Shirzadi, Mohammad Asghari-Jafarabadi

**Affiliations:** ^1^Research Center of Psychiatry and Behavioral Sciences, Tabriz University of Medical Sciences, Tabriz, Iran; ^2^Health Education & Promotion Department, Faculty of Health, Tabriz University of Medical Sciences, Tabriz, Iran; ^3^Social Determinants of Health Research Center, Research Institute for Health Development, Kurdistan University of Medical Sciences, Sanandaj, Iran; ^4^Department of Public Health, Faculty of Health, Neyshabur University of Medical Sciences, Neyshabur, Iran; ^5^Cabrini Research, Cabrini Health, Malvern, VIC, 3144, Australia; ^6^School of Public Health and Preventive Medicine, Monash University, Melbourne, VIC, 3004, Australia; ^7^Department of Psychiatry, School of Clinical Sciences, Monash University, Clayton, VIC, 3168, Australia; ^8^Road Traffic Injury Research Center, Tabriz University of Medical Sciences, Tabriz, Iran

## Dear Editor,

 We are writing to formally submit a corrigendum for our article titled “Medications Non-adherence Reasoning Scale (MedNARS): Development and Psychometric Properties Appraisal,” published in Health Promotion Perspectives on September 11, 2023. 1

 We have identified an error in the Ethical Approval section of the manuscript. The original article inadvertently listed the approval code as IR.TBZMED.REC.1396.435, whereas the correct code should be IR.TBZMED.REC.1399.499.

## Ethical Approval

 Ethical approval for the study protocol was obtained from the Medical Ethics Board of Trustees (MEBoT) affiliated with the Tabriz University of Medical Sciences (IR.TBZMED.REC.1399.499), and all the participants voluntarily signed an informed consent form to participate in the study before the interviews and data collection stage.

 We confirm that this correction applies only to the ethical approval documentation and does not affect the study’s methodology, results, conclusions, or scientific validity.
