# Supplementary material for: Medications Non-adherence Reasoning Scale (MedNARS): Development and psychometric properties appraisal
Source: Health Promot Perspect. 2023 Sep 11;13(3):212–8. doi: 10.34172/hpp.2023.26 (PMC10558970; doi:10.34172/hpp.2023.26)
Supplement: Supplementary file 1 — The Final Medications Non-adherence Reasoning Scale (MedNARS) and scoring guideline. [file hpp-13-212-s001.pdf]

Allahverdipour et al, **Health Promotion Perspectives,** 2023, 13(3), S1.
doi: 10.34172/hpp.2023.26
https://hpp.tbzmed.ac.ir

Supplementary file 1. The Final Medications Non-adherence Reasoning Scale (MedNARS) and scoring guideline

Item	Item content
1	Have you ever stopped taking your prescribed medications due to experiencing their side effects?
2	Have you ever stopped taking your medications due to prescription of their long-term use?
3	Have you ever stopped taking your prescribed medications due to ineffectiveness of your therapeutic regimen?
4	Have you ever stopped taking your medications due to prescription of multiple drug use at the same time?
5	Have you ever stopped taking your prescribed medications due to their interactions?
6	Have you ever stopped taking your prescribed medications due to their taste, size or form?
7	Have you ever stopped taking your prescribed medications due to your illness severity or symptoms?
8	Have you ever stopped taking your prescribed medications due to the impacts of your illness on your life?
9	Have you ever stopped taking your medicines due to being prescribed with multiple medications?

The MedNARS scoring guideline: The MedNARS measures Medication Adherence through items with a 5-point Likert scale, 1 = frequently, 2 = usually, 3 = occasionally, 4 = rarely, and 5 = not at all, so higher scores represent higher levels of Medication Adherence.
